# Effect of eucalyptus oil on *Streptococcus mutans* and *Enterococcus faecalis* growth

**DOI:** 10.1038/s41405-023-00154-8

**Published:** 2023-07-06

**Authors:** Abdulrahman A. Balhaddad, Rasha N. AlSheikh

**Affiliations:** Department of Restorative Dental Sciences, College of Dentistry, Imam Abdulrahman Bin Faisal University, P.O.Box 1982, Dammam, 31441 Saudi Arabia

**Keywords:** Dental biofilms, Preventive dentistry

## Abstract

**Objectives:**

There is a significant apprehension in medicine and dentistry concerning the emergence of antibiotic-resistant pathogens, as it composes a significant threat to global health, particularly oral health. The growing concern that oral pathogens may develop resistance against standard preventive measures raises the need for alternative measures to prevent these pathogens’ growth without inducing microbial resistance. Therefore, this study aims to assess the antibacterial properties of eucalyptus oil (EO) against two main oral disease pathogens, *Streptococcus mutans*, and *Enterococci faecalis*.

**Methods:**

*S. mutans* and *E. faecalis* biofilms were initiated using brain-heart infusion (BHI) broth supplemented with 2% sucrose with and without diluted EO. After 24 h of biofilm formation, total absorbance was measured via spectrophotometer; then, the biofilm was fixed, stained with crystal violet dye, and measured at 490 nm. An Independent *t*-test was used to compare the outcomes.

**Results:**

Diluted EO revealed significant total absorbance reduction against *S. mutans* and *E. faecalis* compared to the control (*p* ≤ 0.001). For the biofilm measurement, *S. mutans* and *E. faecalis* biofilms were reduced by around 60- and 30-fold, respectively, compared to the group with no EO (*p* ≤ 0.001).

**Conclusion:**

Based on this study’s results, using EO as an organic compound could be considered an adjunctive tool in preventing the growth of oral pathogens causing dental caries and endodontic infection.

## Introduction

Dental caries is a prominent dental issue that affects almost all adults worldwide [1, 2]. It is defined as a multifactorial biofilm-triggered disease that demineralizes the tooth structure through repetitive cycles of bacterial-induced acid attack, leading to mineral loss and tooth destruction [1]. Dental caries could be prevented and intervened in the early stages before the physical tooth destruction via non-invasive approaches [2]. One of the effective approaches in controlling caries progression involves using chlorohexidine-containing products [2]. However, there is a significant concern as cariogenic pathogens may develop resistance against chlorohexidine [3]. Therefore, inventing new approaches to target dental pathogens and biofilms without inducing microbial resistance is essential.

Untreated dental caries may allow the involved microorganisms to extend to the pulp, causing inflammation and periapical infection [4]. In such consequences, root canal treatment (RCT) that involves cleaning the root canal system mechanically and chemically is indicated [4, 5]. *Enterococcus faecalis* (*E. faecalis*) is among the most detected bacteria in periapical lesions [6]. *E. faecalis* is associated with failed RCT and endodontic reinfection, which could be attributed to its capabilities to resist antimicrobial medicaments and survive in extreme conditions, such as high pH environments [7, 8]. To prevent root canal reinfection, targeting this microorganism, as well as other species, during chemical disinfection is critical to ensure sterile canals before the obturation [7, 8]. Therefore, designing adjunctive approaches to improve the efficiency of root canal disinfection is highly needed [9].

Several therapeutic approaches have been proposed to control dental caries and endodontic infections [10, 11]. While the two diseases have different clinical management, using some medicaments, such as chlorohexidine or antibiotics, has been suggested to disinfect dental tissues for both diseases [10, 11]. The main concern related to using these medicaments is the possibility of inducing bacterial resistance [12]. It was found that around 25,000 deaths yearly are recorded due to antibiotic-resistant diseases in the European Union [13]. As a result, exploring alternative approaches to intervene with oral infections with minimum risk of inducing bacterial resistance is essential to advance oral health care practice with minimum adverse effects [14].

Throughout the history of humankind, several herbal and natural compounds have been utilized to cure diseases and kill microbes [15]. However, the evidence for using these compounds still needs to be more substantial and worthy of being discovered. A plethora of evidence supports using essential oils as antimicrobial compounds to kill viruses, fungi, and bacteria [16]. Among them, eucalyptus oil (EO) was barely explored for its antimicrobial properties. Therefore, the antibiofilm properties of EO against *Streptococcus mutans* (*S. mutans*), one of the main players in dental caries pathogenesis, and *E. faecalis* are worth evaluating.

*S. mutans* plays a major role in the initiation and progression of dental caries, mainly due to its ability to form a biofilm and provide a habitat for other cariogenic bacteria to flourish and adhere to tooth structures by producing multiple binding proteins [17]. On the other hand, *E. faecalis* represents the most isolated microorganism (45.8%) from endodontic failures and is characterized by being the most persistent in endodontic disinfection measures [6, 18]. The overall antibacterial properties’ assessment of EO against these two microorganisms will provide an idea concerning the potential use of this organic compound to prevent dental caries and endodontic reinfection. Therefore, this study hypothesizes that EO would inhibit the growth of *S. mutans* and *E. faecalis*.

## Methodology

### Study design

The Research Unit at Imam Abdulrahman bin Faisal University (IAU) approved this study, confirming ethical approval was not needed. Eucalyptus oil (Spectrum, New Brunswick, NJ) was mixed with brain-heart infusion (BHI) broth supplemented with 2% sucrose at a 1:5 ratio. The mixture was used immediately after mixing. The study had two groups; EO dilution (experimental) and BHI supplemented with 2% sucrose as a control. Both experimental and control groups were incubated with an overnight culture of *S. mutans* and *E. faecalis* strains for 24 h at a ratio of 20:1.

### Experimental setting

**Fig. 1:**
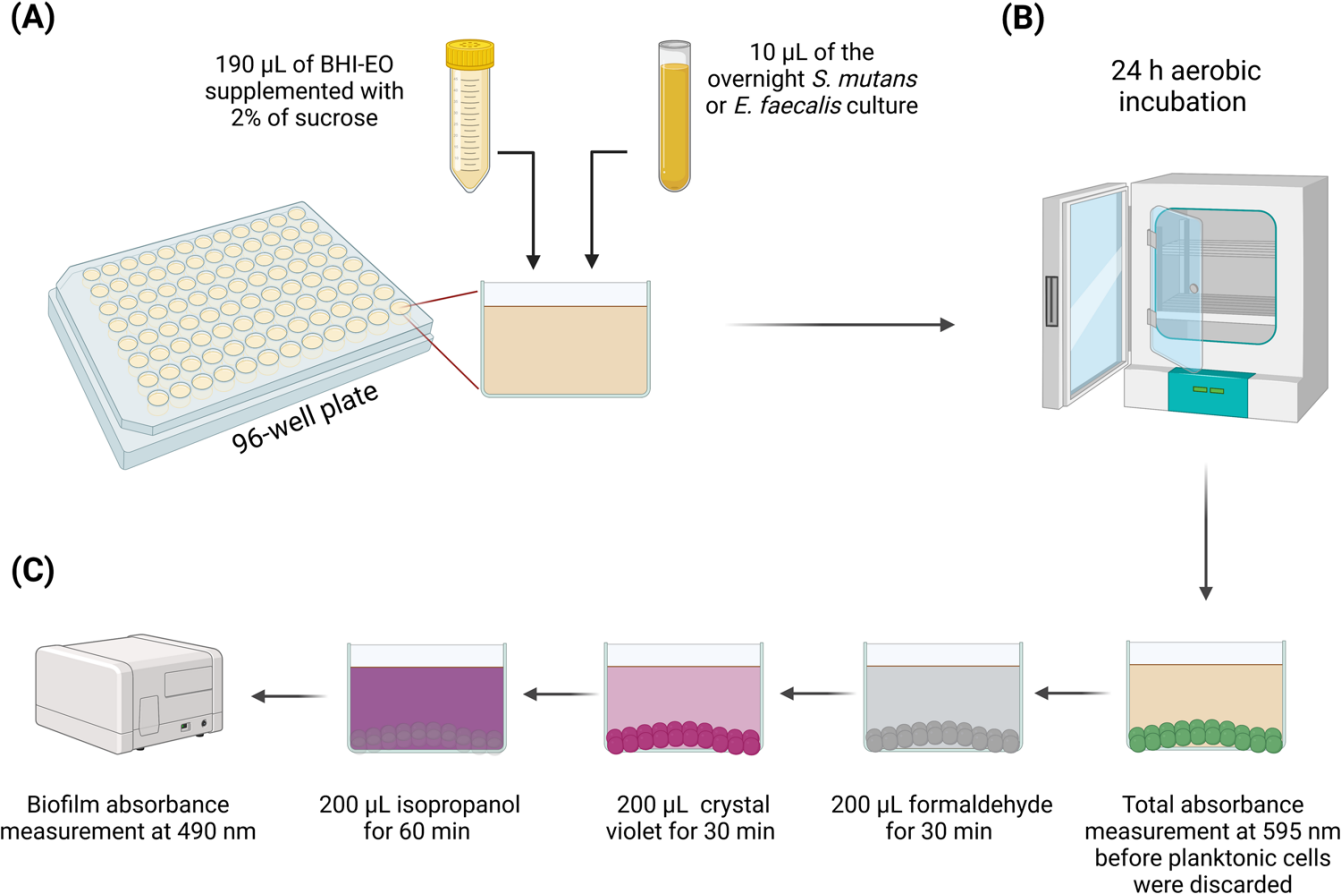
Schematic drawing illustrating the design of the study. *Streptococcus mutans* and *Enterococcus faecalis* were grown overnight in brain-heart infusion (BHI) broth. **A** 10 µL of the overnight culture of each strain was added in a 96-well plate with 190 µL of fresh BHI broth supplemented with 2 wt.% of sucrose with diluted eucalyptus oil (BHI-EO) or with no eucalyptus oil as a control. **B** The 96-well plate was incubated aerobically for 24 h at 5% CO_2_. **C** The following day, the total and biofilm absorbance was read at 595 and 490 nm, respectively. Created with BioRender.com

*S. mutans* (UA159) and *E. faecalis* (ATCC29212) cultures were grown overnight using BHI broth. The optical density was adjusted to be 0.9 and 1.0 for *S. mutans* and *E. faecalis*, respectively. Then, 10 µL of the overnight *S. mutans* or *E. faecalis* cultures were added to 190 µL of the EO dilution (EO + BHI supplemented with 2% of sucrose) or BHI supplemented with 2% of sucrose (control) inside the wells of a 96-well plate (Fig. 1A) [19]. A 24 h incubation was achieved using an aerobic incubator at 5% CO_2_. The following day, the total absorbance (planktonic and biofilm) was measured at 595 nm via a spectrophotometer (SpectraMax M5, Molecular Devices, Sunnyvale, CA, USA). Then, the planktonic cells were discarded, and the attached biofilm was treated with 200 µL of 10% formaldehyde for 30 min. Then, three times deionized water washing was achieved to remove the formaldehyde, and the biofilm was stained using 200 µL of 0.5% crystal violet dye for 30 min. The wells were washed thrice to remove the crystal violet stain, keeping only the stained biofilms, and 200 µL of 2-isopropanol was added for 1 h to lyse the cells (Fig. 1C) [15, 19, 20]. The spectrophotometer measurement at 490 nm was achieved to quantify the biofilm absorbance.

### Statistical analysis

Sigma plot recorded and analyzed the data. Descriptive statistics (mean, standard deviation, frequency, and percentages) were used to summarize the information, and the Shapiro-Wilk test was used to test data normality. An Independent *t*-test was used to compare the outcomes. A *P* value of <0.05 was considered statistically significant.

## Results

**Fig. 2:**
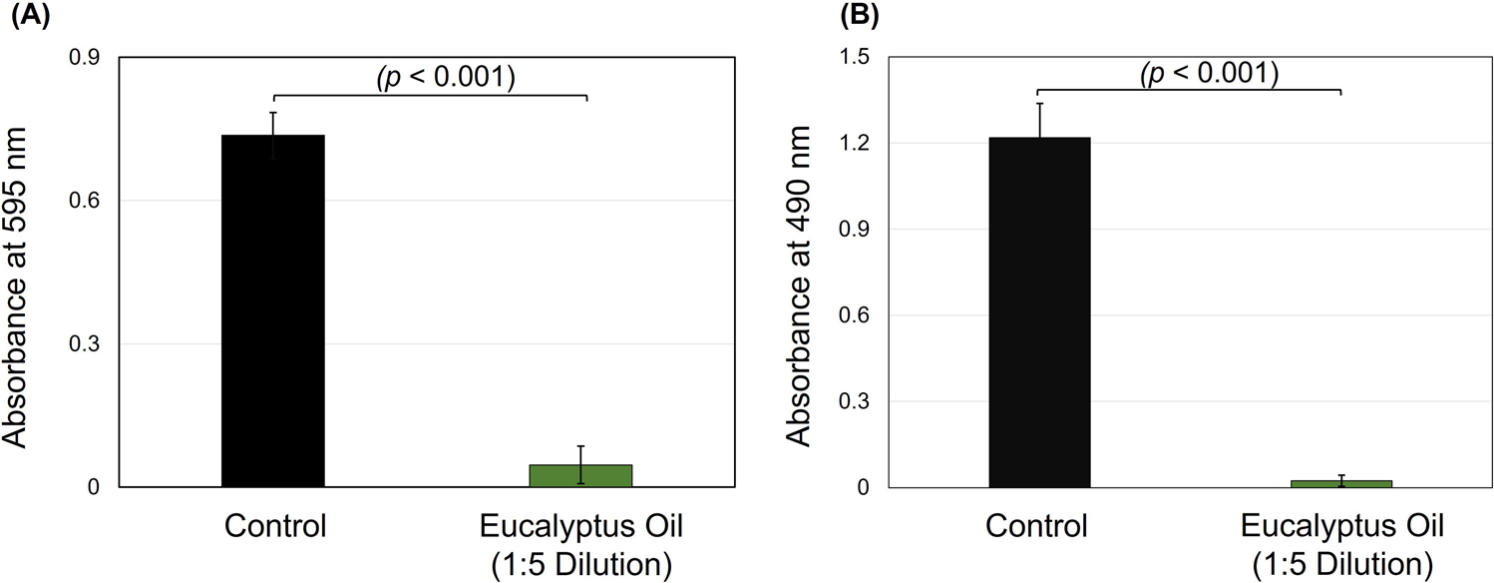
The antibacterial activities of eucalyptus oil against *Streptococcus mutans.* Eucalyptus oil significantly reduced the total absorbance (**A**) and biofilm formation (**B**) growth of *S. mutans*.

Following the 24 h *S. mutans* growth, samples (*n* = 12) treated with eucalyptus oil dilution revealed significant total absorbance reduction compared to the control (*p* < 0.001, power of analysis = 100%). The mean average of the control samples’ total absorbance (Fig. 2A) was 0.74 compared to 0.05 for those treated with eucalyptus oil dilution. Similarly, the eucalyptus oil significantly inhibited the *S. mutans* biofilm growth (Fig. 2B) when it was incubated for 24 h (*p* < 0.001, power of analysis = 100%). The average value of these wells treated with oil was 1.22, compared to 0.02 for the control with no oil treatment. These results indicate that eucalyptus oil as an organic compound can be used to prevent caries-related pathogens’ growth.

**Fig. 3:**
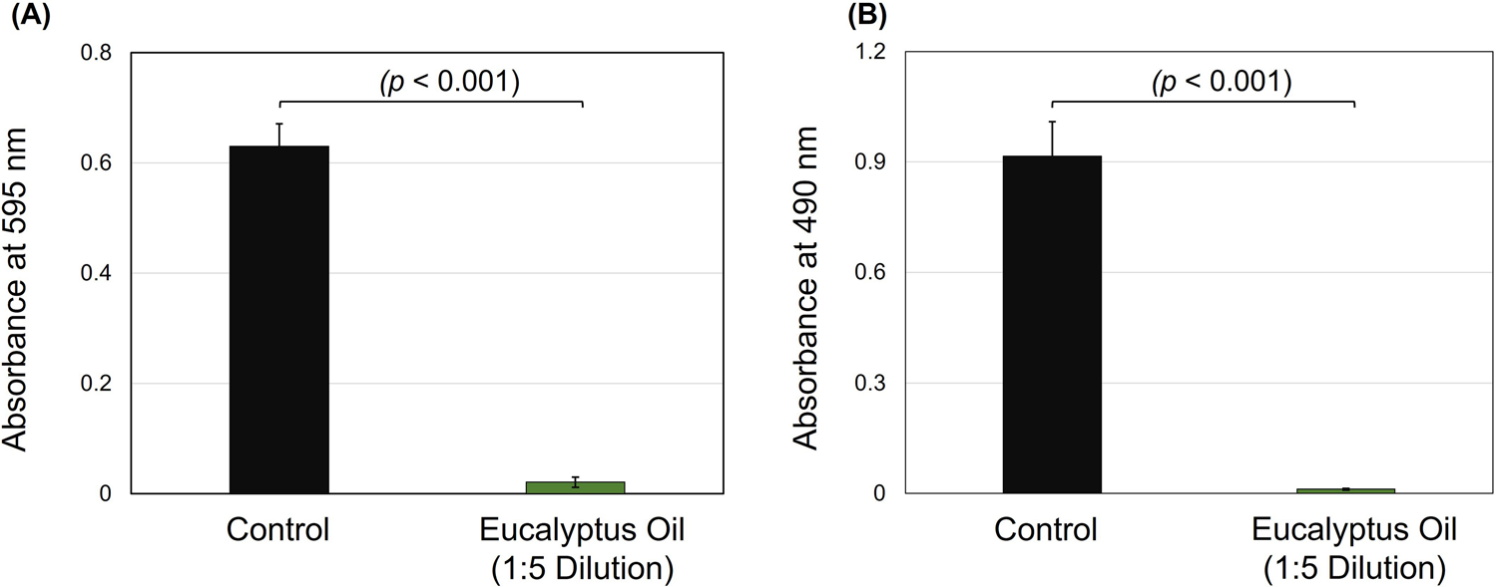
The antibacterial activities of eucalyptus oil against *Enterococcus faecalis*. Eucalyptus oil significantly reduced the total absorbance (**A**) and biofilm formation (**B**) growth of *E. faecalis*.

For *E. faecalis*, EO achieved significant growth inhibition (*n* = 12). The total absorbance growth was reduced by more than 30-fold compared to the control (*p* < 0.001, power of analysis = 100%). The mean average of the control samples’ total absorbance (Fig. 3A) was 0.63 compared to 0.02 for those treated with EO dilution. For the biofilm reading, EO could inhibit the *E. faecalis* biofilm by around 30-fold (*p* < 0.001, power of analysis = 100%). The average value of these wells treated with oil was 0.91, compared to 0.01 for the control with no treatment.

## Discussion

This study investigated the antibacterial effect of *Eucalyptus oil* (EO) against *S. mutans* and *E. faecalis* for potentially using it as an adjunctive approach to standard preventive measures in controlling dental caries and endodontic infection. The results demonstrated here found that EO significantly inhibited the planktonic and biofilm growth of *S. mutans* and *E. faecalis*. These findings agree with published literature [21–24] that illustrated the capabilities of other essential oils, such as *Eucalyptus globulus* and *Mentha spicata*, in inhibiting *S. mutans* and *E. faecalis* growth. In one study, around 100 µL of several essential oils was placed in blood agar plates and inoculated with 100 µL of *S. mutans* culture. Essential oils such as *Cinnamon*, *Lemongrass*, *Cedarwood*, *Clove*, and *eucalyptus oils* revealed 3.44 to 12.51 mm of zone inhibition against *S. mutans* growth [25], which is found to be exceeding the chlorohexidine inhibition zone of 2.83 ± 0.36 mm [26]. However, in the same study, *Wintergreen*, *Lime*, *Spearmint*, and *Peppermint oils* were found ineffective [25], indicating that essential oils may differ concerning their antimicrobial properties.

One of the limitations in the previous study was testing essential oils against the bacterial species in their planktonic forms [25]. It is well-known that microbial species in their biofilm forms are more resistant to therapeutic agents [27]. The biofilm matrix and its associated structure can limit the diffusion and permeability of antimicrobial agents toward the core of biofilms, allowing the embedded bacteria to survive and develop certain resistance mechanisms against such agents [27]. Therefore, it is necessary to challenge and test the antimicrobial properties of such materials against the biofilm form of the tested microorganisms. In this study, the antibacterial effect of EO against the planktonic and biofilm growth of *S. mutans* and *E. faecalis* was prominent, indicating its potential use against thick and mature dental biofilms.

The efficiency of essential oils against *S. mutans* biofilms was also evaluated in another study, where *Eucalyptus globulus* and *Eucalyptus urograndis* demonstrated antibiofilm inhibition against 48-h *S. mutans* biofilm [28]. In a recent investigation, *Mentha spicata* and *Eucalyptus globulus* inhibited *S. mutans* colony-forming units (CFUs) by around 1 to 1.5-fold, respectively [23]. Besides, other cariogenic species, such as *Streptococcus sanguis* and *Streptococcus salivarius*, were inhibited by different essential oils [22]. The capabilities of essential oils to inhibit *E. faecalis* were also investigated. A 5-log reduction of *E. faecalis* growth was observed when this bacterium was treated with *Eucalyptus globulus* [24]. More interestingly, when essential oils were tested against multi-drug-resistant *E. faecalis*, *Origanum glandulosum* and *Thymbra capitata* were found highly effective in eradicating the *E. faecalis* biofilms [29]. Such findings highlight the potent effect of essential oils and their capabilities to manage recurrent infections that could be enriched with drug-resistant microorganisms.

In this study, the antibiofilm effect of EO was the focus, as this oil was barely examined for its antibiofilm action against oral pathogens. EO inhibited the biofilm growth of *S. mutans* and *E. faecalis* by around 60 and 30-fold, respectively, compared to the group with no EO. It has been suggested that 1,8-cineole, the major component of EC, is the potent antimicrobial ingredient, as it has been found effective against many bacteria, fungi, and viruses [30]. One study revealed that the higher the 1,8-cineole concentration, the greater the antimicrobial action [28]. 1,8-cineole can induce oxidative stress, causing bacterial membrane damage and leakage of the intercellular components [31]. Such observations might suggest that instead of using essential oils to target dental microbes, 1,8-cineole itself could be isolated and functionalized for the intended clinical use. This may maximize the antimicrobial action of the designed drug, resulting in more significant biofilm inhibition and more clinical benefits.

Besides its antimicrobial action, EO can improve the efficiency of other conjugated antibacterial compounds, allowing more biofilm penetration and inhibition [19]. It has been suggested that EO can allow better membrane permeability for the conjugated antimicrobial agents, which can facilitate targeting intracellular components of such microorganisms [19]. All these observations encourage the use of EO as a natural therapeutic approach in dentistry to replace the current treatment modalities that may induce microbial resistance.

Currently, most over-the-counter mouthwashes contain alcohol. Despite the controversy that alcoholic mouthwashes may cause oral cancer [32], having a natural alternative with a low risk of irritating the oral soft tissues and inducing bacterial resistance will be highly beneficial to the field of preventive dentistry. In pharmacies, many oral health products contain oil derivatives and herbal compounds as bioactive ingredients to prevent dental caries [33, 34]. The incorporation of herbal compounds in oral health products was examined in several investigations. For instance, Rasooli et al. investigated the effect of experimental toothpaste containing *Eucalyptus Camalduensis* oil against *S. mutans* with a particular focus on in vivo and in vitro biofilm formation [35]. They stated that the oil exerted an antimicrobial effect against the tested microorganisms exceeding that of chlorhexidine significantly (*p* < 0.001) [35]. Ravi et al. evaluated the zone of inhibition and determined the minimum inhibitory concentration (MIC) of *Mango* and *Eucalyptus twig* extracts on *S. mutans* and the possibility of utilizing the oil in dentifrices. The *Mango* extract showed a significantly higher antibacterial effect at a lower concentration, while *E. twig* oil showed a higher percentage of inhibition at 90.9% [36].

Similarly, EO can be cost-effectively incorporated into different oral health products, such as toothpaste and mouthwash, to minimize cariogenic species’ load and reduce dental caries’ incidence. Besides, EO can be functionalized into a dental varnish or topically applied agent to arrest non-cavitated carious lesions and replace some treatment modalities that might induce bacterial resistance.

In root canal therapy, periapical reinfection is a significant challenge in the endodontic field. While using sodium hypochlorite to disinfect the root canal system is the gold standard approach, complete disinfection can not be guaranteed [9]. As a result, adjunctive approaches such as photodynamic therapy and applying organic and inorganic compounds were attempted [37, 38]. Our study’s results may suggest using EO as an adjunctive approach to improving the disinfection protocol of the root canal system, which may minimize the onset of endodontic reinfection. This suggestion could be supported by recent investigations indicating the capabilities of essential oils, such as *Chamomile* oils [39] and *Cymbopogon martini* [40], to improve the disinfection of the root canal system ex vivo. Besides, essential oils may provide anti-inflammatory action, improving the healing process in the periapical region [41]. The initial results described in this study may encourage designing an ex vivo endodontic infection model, where EO could be used as irrigation. In such a model, EO could be used alone as an irrigation solution or combined with sodium hypochlorite to explore potential synergetic effects.

The results of this study should be interpreted carefully, as the use of conventional preventive and therapeutic approaches must still be the gold standard in preventing biofilm-triggered diseases. With the limitation of this study, more quantitative testing is needed to estimate the dose and the application method to use EO to prevent dental caries and endodontic reinfections. Furthermore, in vivo studies will be beneficial to evaluate the use of EO inside the oral cavity, where the complexity of the oral biofilms and the influence of some host-related factors can also be tested. Future investigations may consider attempting EO against other oral pathogens, such as periodontal pathogens and different *Candida* species.

## Conclusion

This study found that using EO in 1:5 dilution effectively reduced the biofilm formation of two dental pathogens, *S. mutans* and *E. faecalis*. Such findings may suggest using EO to intervene with dental caries and endodontic infection with a minimum risk of microbial resistance induction. Future studies may implement a clinical translational model to investigate the antibacterial effect of EO inside the oral environment.

## Data Availability

The data published in this paper is available upon request.

## References

[1] Lemos JA, Palmer SR, Zeng L, Wen ZT, Kajfasz JK, Freires IA, et al. The biology of *Streptococcus mutans*. Microbiol Spectr. 2019;7. 10.1128/microbiolspec.GPP3-0051-2018 10.1128/microbiolspec.gpp3-0051-2018 PMC6615571 30657107

[2] Balhaddad AA, Kansara AA, Hidan D, Weir MD, Xu HHK, Melo MAS (2019). Toward dental caries: exploring nanoparticle-based platforms and calcium phosphate compounds for dental restorative materials. Bioact Mater.

[3] Kampf G (2016). Acquired resistance to chlorhexidine - is it time to establish an “antiseptic stewardship” initiative?. J Hosp Infect.

[4] Siqueira JF, Rôças IN (2022). Present status and future directions: microbiology of endodontic infections. Int Endod J.

[5] Alquria TA, Alfirdous RA, Gupta S, Santamaria MP, Santamaria IF, Gomes APM (2023). Comparison of conventional and contemporary root canal disinfection protocols against bacteria, lipoteichoic acid (LTA), and lipopolysaccharide (LPS). Sci Rep.

[6] Prada I, Micó-Muñoz P, Giner-Lluesma T, Micó-Martínez P, Collado-Castellano N, Manzano-Saiz A (2019). Influence of microbiology on endodontic failure. Literature review. Med Oral Patol Oral Cir Bucal.

[7] Francisco PA, Fagundes PI, da G, Lemes-Junior JC, Lima AR, Passini MRZ (2021). Pathogenic potential of *Enterococcus faecalis* strains isolated from root canals after unsuccessful endodontic treatment. Clin Oral Investig.

[8] Stuart CH, Schwartz SA, Beeson TJ, Owatz CB (2006). Enterococcus faecalis: its role in root canal treatment failure and current concepts in retreatment. J Endod.

[9] Alfirdous RA, Garcia IM, Balhaddad AA, Collares FM, Martinho FC, Melo MAS (2021). Advancing photodynamic therapy for endodontic disinfection with nanoparticles: present evidence and upcoming approaches. Appl Sci.

[10] Warreth A (2023). Dental caries and its management. Int J Dent.

[11] Segura-Egea JJ, Gould K, Şen BH, Jonasson P, Cotti E, Mazzoni A (2017). Antibiotics in endodontics: a review. Int Endod J.

[12] Qiu W, Zhou Y, Li Z, Huang T, Xiao Y, Cheng L (2020). Application of antibiotics/antimicrobial agents on dental caries. Biomed Res Int.

[13] Alós J-I (2015). [Antibiotic resistance: a global crisis]. Enferm Infecc Microbiol Clin.

[14] Brooks L, Narvekar U, McDonald A, Mullany P (2022). Prevalence of antibiotic resistance genes in the oral cavity and mobile genetic elements that disseminate antimicrobial resistance: a systematic review. Mol Oral Microbiol.

[15] Balhaddad AA, Mokeem L, Melo MAS, Gregory RL (2021). Antibacterial activities of methanol and aqueous extracts of salvadora persica against *Streptococcus mutans* biofilms: an in vitro study. Dent J.

[16] Bassolé IHN, Juliani HR (2012). Essential oils in combination and their antimicrobial properties. Molecules.

[17] Yamashita Y, Bowen WH, Burne RA, Kuramitsu HK (1993). Role of the *Streptococcus mutans* gtf genes in caries induction in the specific-pathogen-free rat model. Infect Immun.

[18] Mohammadi Z, Palazzi F, Giardino L, Shalavi S (2013). Microbial biofilms in endodontic infections: an update review. Biomed J.

[19] Balhaddad AA, Xia Y, Lan Y, Mokeem L, Ibrahim MS, Weir MD (2021). Magnetic-responsive photosensitizer nanoplatform for optimized inactivation of dental caries-related biofilms: technology development and proof of principle. ACS Nano.

[20] Balhaddad AA, Melo MAS, Gregory RL. Inhibition of nicotine-induced *Streptococcus mutans* biofilm formation by salts solutions intended for mouthrinses. Restor Dent Endod. 2019;44. 10.5395/rde.2019.44.e4 10.5395/rde.2019.44.e4 PMC6387890 30834226

[21] Chandra Shekar BR, Nagarajappa R, Jain R, Singh R, Thakur R, Shekar S (2016). Antimicrobial efficacy of Acacia nilotica, Murraya koenigii (L.) Sprengel, Eucalyptus hybrid, Psidium guajava extracts and their combination on Streptococcus mutans and Lactobacillus acidophilus. Dent Res J.

[22] Shekar C, Nagarajappa R, Singh R, Thakur R (2015). Antimicrobial efficacy of Acacia nilotica, Murraya koenigii L. Sprengel, Eucalyptus hybrid, and Psidium guajava on primary plaque colonizers: An in vitro comparison between hot and cold extraction process. J. Indian Soc. Periodontol.

[23] Landeo-Villanueva GE, Salazar-Salvatierra ME, Ruiz-Quiroz JR, Zuta-Arriola N, Jarama-Soto B, Herrera-Calderon O (2023). Inhibitory activity of essential oils of mentha spicata and eucalyptus globulus on biofilms of *Streptococcus mutans* in an in vitro model. Antibiotics.

[24] Ambrosio CMS, de Alencar SM, Moreno AM, Da Gloria EM (2018). Evaluation of the selective antibacterial activity of Eucalyptus globulus and Pimenta pseudocaryophyllus essential oils individually and in combination on Enterococcus faecalis and Lactobacillus rhamnosus. Can J Microbiol.

[25] Chaudhari LKD, Jawale BA, Sharma S, Sharma H, Kumar CDM, Kulkarni PA (2012). Antimicrobial activity of commercially available essential oils against *Streptococcus mutans*. J Contemp Dent Pract.

[26] Alauddin MS, Yusof NM, Adnan AS, Said Z (2022). Preliminary novel analysis on antimicrobial properties of concentrated growth factor against bacteria-induced oral diseases. Eur J Dent.

[27] Flemming H-C, Wingender J (2010). The biofilm matrix. Nat Rev Microbiol.

[28] Goldbeck JC, do Nascimento JE, Jacob RG, Fiorentini ÂM, da Silva WP (2014). Bioactivity of essential oils from Eucalyptus globulus and Eucalyptus urograndis against planktonic cells and biofilms of *Streptococcus mutans*. Ind Crops Prod.

[29] Benbelaïd F, Khadir A, Abdoune MA, Bendahou M, Muselli A, Costa J (2014). Antimicrobial activity of some essential oils against oral multidrug–resistant Enterococcus faecalis in both planktonic and biofilm state. Asian Pac Trop Biomed.

[30] Sadlon AE, Lamson DW (2010). Immune-modifying and antimicrobial effects of Eucalyptus oil and simple inhalation devices. Alter Med Rev.

[31] Moo C-L, Osman MA, Yang S-K, Yap W-S, Ismail S, Lim S-H-E (2021). Antimicrobial activity and mode of action of 1,8-cineol against carbapenemase-producing Klebsiella pneumoniae. Sci Rep.

[32] de A Werner CW, Seymour RA (2009). Are alcohol containing mouthwashes safe?. Br Dent J.

[33] Tidke S, Chhabra GK, Madhu PP, Reche A, Wazurkar S, Singi SR (2022). The effectiveness of herbal versus non-herbal mouthwash for periodontal health: a literature review. Cureus.

[34] Alshehri FA (2018). The use of mouthwash containing essential oils (LISTERINE®) to improve oral health: a systematic review. Saudi Dent J.

[35] Rasooli I, Shayegh S, Astaneh S (2009). The effect of Mentha spicata and *Eucalyptus camaldulensis* essential oils on dental biofilm. Int J Dent Hyg.

[36] Banavar Ravi S, Nirupad S, Chippagiri P, Pandurangappa R (2017). Antibacterial effects of natural herbal extracts on *Streptococcus mutans*: can they be potential additives in dentifrices?. Int J Dent.

[37] Raura N, Garg A, Arora A, Roma M (2020). Nanoparticle technology and its implications in endodontics: a review. Biomater Res.

[38] Zorita-García M, Alonso-Ezpeleta LÓ, Cobo M, Del Campo R, Rico-Romano C, Mena-Álvarez J (2019). Photodynamic therapy in endodontic root canal treatment significantly increases bacterial clearance, preventing apical periodontitis. Quintessence Int.

[39] Shakya VK, Luqman S, Tikku AP, Chandra A, Singh DK (2019). A relative assessment of essential oil of Chrysopogon zizanioides and Matricaria chamomilla along with calcium hydroxide and chlorhexidine gel against Enterococcus faecalis in ex vivo root canal models. J Conserv Dent.

[40] Marinković J, Ćulafić DM, Nikolić B, Đukanović S, Marković T, Tasić G (2020). Antimicrobial potential of irrigants based on essential oils of *Cymbopogon martinii* and Thymus zygis towards in vitro multispecies biofilm cultured in ex vivo root canals. Arch Oral Biol.

[41] Marinković J, Nikolić B, Marković T, Petrović B, Pašalić S, Lal M (2022). Essential oils as adjuvants in endodontic therapy: myth or reality?. Future Microbiol.

