# Correction: Effect of eucalyptus oil on *Streptococcus mutans* and *Enterococcus faecalis* growth

**DOI:** 10.1038/s41405-023-00173-5

**Published:** 2023-10-16

**Authors:** Abdulrahman A. Balhaddad, Rasha N. AlSheikh

**Affiliations:** Department of Restorative Dental Sciences, College of Dentistry, Imam Abdulrahman Bin Faisal University, P.O.Box 1982, Dammam, 31441 Saudi Arabia

**Keywords:** Dental biofilms, Preventive dentistry

Correction to: *BDJ Open* 10.1038/s41405-023-00154-8, published online 06 July 2023

When originally published on 6 July 2023, an incorrect ratio was included in the article. Within the ‘Methodology’ section, ‘Eucalyptus oil (Spectrum, New Brunswick, NJ) was mixed with brain-heart infusion (BHI) broth supplemented with 2% sucrose at a **1:4** ratio’ should have read ‘Eucalyptus oil (Spectrum, New Brunswick, NJ) was mixed with brain-heart infusion (BHI) broth supplemented with 2% sucrose at a **1:5** ratio’. Similarly, in the ‘Conclusion’ section, ‘This study found that using EO in **1:4** dilution effectively reduced the biofilm formation of two dental pathogens, *S. mutans* and *E. faecalis*’ should have read ‘This study found that using EO in **1:5** dilution effectively reduced the biofilm formation of two dental pathogens, *S. mutans* and *E. faecalis*’.

Finally, in the Figure 1 caption, the title incorrectly read ‘Schematic drawing illustrating the design of the stud’. This should have read ‘Schematic drawing illustrating the design of the study’.

The authors apologise for any inconvenience caused.

